# The Relationship between Crystal Structure and Mechanical Performance for Fabrication of Regenerated Cellulose Film through Coagulation Conditions

**DOI:** 10.3390/polym13244450

**Published:** 2021-12-18

**Authors:** Tessei Kawano, Satoshi Iikubo, Yoshito Andou

**Affiliations:** 1 Department of Life Science and Systems Engineering, Graduate School of Life Science and Systems Engineering, Kyushu Institute of Technology, 2-4 Hibikino, Wakamatsu, Kitakyushu 808-0196, Fukuoka, Japan; kawano.tessei758@mail.kyutech.jp (T.K.); iikubo@life.kyutech.ac.jp (S.I.); 2 Department of Advanced Materials Science and Engineering, Faculty of Engineering Sciences, Kyushu University, Kasuga 816-8580, Fukuoka, Japan; 3 Collaborative Research Centre for Green Materials on Environmental Technology, Kyushu Institute of Technology, 2-4 Hibikino, Wakamatsu, Kitakyushu 808-0196, Fukuoka, Japan

**Keywords:** regenerated cellulose, alkali–urea solution, coagulation conditions, crystal structure

## Abstract

Cellulose films regenerated from aqueous alkali–urea solution possess different properties depending on coagulation conditions. However, the correlation between coagulant species and properties of regenerated cellulose (RC) films has not been clarified yet. In this study, RC films were prepared from cellulose nanofiber (CNF) and microcrystalline cellulose (MCC) under several coagulation conditions. Cellulose dissolved in aqueous LiOH–urea solution was regenerated using various solvents at ambient temperature to investigate the effects of their dielectric constant on the properties of RC film. The crystal structure, mechanical properties, and surface morphology of prepared RC films were analyzed using X-ray diffraction (XRD), tensile tester, and atomic probe microscopy (AFM), respectively. It is revealed that the preferential orientation of (110) and (020) crystal planes, which are formed by inter- and intramolecular hydrogen bonding in cellulose crystal regions, changed depending on coagulant species. Furthermore, we found out that tensile strength, elongation at break, and crystal structure properties of RC films strongly correlate to the dielectric constant of solvents used for the coagulation process. This work, therefore, would be able to provide an indicator to control the mechanical performance of RC film depending on its application and to develop detailed researches on controlling the crystal structure of cellulose.

## 1. Introduction

The development of a replacement for petroleum-based plastics is becoming more necessary in the face of global warming and plastic pollution. In particular, single-use plastics (SUPs), such as plastic bags, packaging materials, and straws, are a substantial source of plastic pollution [1]. In recent years, many types of bio-based and biodegradable plastics have been developed. For instance, polylactic acid (PLA) is one of the most widely used biodegradable polymers, and PLA-based nanocomposites have attracted interest in a variety of applications [2,3,4,5,6]. Besides, polyhydroxyalkanoates (PHAs) synthesized naturally by various microorganisms have also been expected as an alternative to petroleum-based plastics [7,8,9]. However, these polymers have limited thermal stability, mechanical strength, and low degradation rate [10,11,12]. In addition to biodegradable polymers, polymer materials filled with lignocellulosic materials as renewable reinforcements have also been studied extensively [13,14,15,16,17]. Despite the significant advantages of lignocellulosic materials as filler, these polymer composites cannot still be considered sustainable materials.

Regenerated cellulose (RC) film, which is composed solely of cellulose, has attracted attention as an environmentally friendly material, especially in food and medicine packaging application due to its biodegradability, gas barrier property, and high mechanical strength [18,19,20]. As cellulose does not melt unlike the common thermoplastics due to the strong hydrogen bonding interactions and physical entanglement, RC film is generally prepared through dissolution, coagulation, and drying processes. Various solvent systems for the dissolution of cellulose have been reported throughout the years, such as carbon disulfide (CS_2_) [21], *N*-methylmorpholine-*N*-oxide (NMMO) [22,23,24], and ionic liquids (ILs) [25,26,27,28,29,30]. Among these solvent systems, aqueous alkali–urea solution has received considerable attention, owing to its simplicity, low toxicity, and rapid dissolution. This solvent system can rapidly dissolve cellulose at low temperatures without producing any hazardous byproducts and significant degradation of cellulose [31]. It has also been reported that the solubility of cellulose in LiOH–urea aqueous solution is higher than that of NaOH–urea solution [32]. In alkali–urea aqueous solution, after ^15^N and ^23^Na NMR measurements were clarified, adding urea can effectively improve the stability of the alkali–cellulose complex, owing to the strong interaction between alkali hydroxides and urea, and low temperature can accelerate the breakdown of intermolecular hydrogen bonding among cellulose and, thus, prevent the agglomeration of alkali–urea–cellulose inclusion complexes [33].

In general, coagulation–regeneration of cellulose from alkali–urea solution is carried out with an acidic aqueous solution or polar organic solvents, and it has been discovered that the properties of RC materials, such as crystal structure, mechanical properties, and surface wettability, can widely vary with types of coagulant and coagulation conditions [34,35,36,37]. For instance, Zhang et al. have undertaken a detailed study on the effects of coagulation conditions of H_2_SO_4_ aqueous solution and revealed that the tensile strength of RC film increases slightly as the H_2_SO_4_ concentration and coagulation time increase, but a continuing increase in the concentration and time causes a decrease in tensile strength [38]. Besides aqueous solution, the effects of organic coagulants, such as methanol, ethanol, and acetone, on the properties of regenerated cellulose have also been discussed. Isobe et al. have reported that the pore size distribution of regenerated cellulose hydrogel is not affected by the type of coagulant, while regenerated cellulose gel coagulated using the aqueous solution and organic solvents show different surface morphology and wettability because of the changes in the crystal structure [39]. Up to now, the effects of coagulation conditions have been widely discussed. Nevertheless, there is currently no literature that clarifies the correlation between the properties of RC films and the types of coagulants. A central question to reveal this correlation would be how the crystal structure of cellulose changes depending on coagulant species during the regeneration process, and how the changes in crystal structure affect the mechanical strength and flexibility of RC films. It is well known that RC has a crystal structure of cellulose II composed of three crystal planes, which are (1–10), (110), and (020) planes [40]. In addition, Yamane et al. revealed that (110) and (020) were formed by the arrangement of the glucan chains via inter-and intra- hydrogen bonding, while the planar glucan chains were arranged through weak hydrophobic interactions in (1–10) plane. Based on this knowledge, we presumed the polarity of coagulant would affect the interactions among cellulose during the regeneration process, and so the composition of crystal planes, leading to the changes in mechanical properties of RC films, the composition of these three crystal planes, and, thus, the mechanical strength of RC films. Therefore, the purpose of the present contribution is to investigate the crystal structure, especially the composition of crystal planes and mechanical properties of RC films, which were prepared through various coagulation conditions, and to clarify and visualize the correlation between characteristics of RC films and types of coagulant. 

In this study, RC films were prepared from cellulose dissolved into LiOH–urea aqueous solution and coagulated using the acid aqueous solution and organic solvents, which have different polarities represented by the dielectric constant. The crystal structure of prepared RC films was analyzed using X-ray diffraction (XRD) and we successfully correlated the changes in the composition of crystal planes with the polarity of coagulants. In addition, it is revealed that the occupancy of (110) and (020) crystal planes which are formed by strong hydrogen bonding is one of the factors that change the mechanical properties of RC films. This study suggests the detailed researches on tuning the performance of RC films through its crystal structure, and provide an indicator to control the properties of RC film depending on its application.

## 2. Materials and Methods

### 2.1. Materials

The water slurry of cellulose nanofiber (CNF) with a concentration of 5.0 wt% and the degree of polymerization (DP) of 650 was provided by Sugino Machine Limited (Toyama, Japan), and stored in a refrigerator before use. Microcrystalline cellulose (MCC) with a particle size of 20 μm and lithium hydroxide (LiOH, ≥98%) were purchased from Sigma-Aldrich (Tokyo, Japan). Urea and all solvents were supplied from Wako Pure Chemical Corporation (Osaka, Japan). All reagents and solvents were laboratory grade and were used as received.

### 2.2. Preparation of Regenerated Cellulose Films

**Figure 1:**
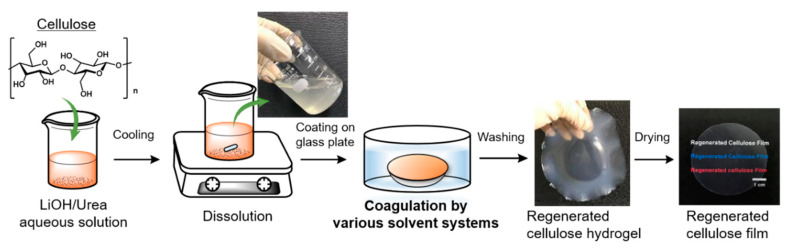
Schematic representation of the RC film preparation.

**Figure 2:**
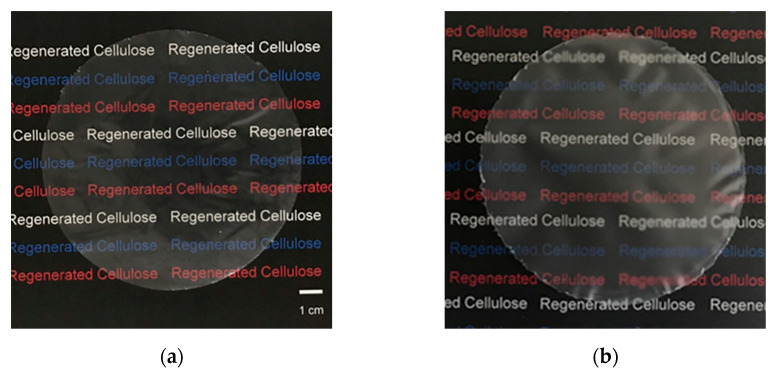
Images of regenerated cellulose film prepared from (**a**) 3 wt% CNF and (**b**) 9 wt% MCC solution in LiOH–urea–H_2_O and coagulated with methanol.

In this study, regenerated cellulose (RC) films were prepared following a slightly modified procedure of the LiOH–urea aqueous system reported elsewhere [41]. Each preparation step is illustrated in Figure 1. The solvent mixture comprising LiOH, urea, and H_2_O with a weight ratio of 4.6:15:80.4, containing the desired amount of cellulose, was cooled at −14 °C for an hour. The concentration of CNF was fixed at 3 wt%. The cooled mixture was then vigorously stirred for 30 min. Cooling and stirring processes were repeated at least twice to make the cellulose completely dissolve in LiOH–urea aqueous solution. The resulting transparent and viscous solution was degassed by centrifugation at 5 °C and 8500 rpm for 3 min, spread on a glass plate using a spin coating apparatus, and immersed in a coagulation bath at ambient temperature. Various types of solvents with different polarity were adopted as a coagulant. The coagulation conditions, including coagulation time and dielectric constant of each solvent, are shown in Table 1. The coagulation by 5 wt% H_2_SO_4_ aqueous solution containing 5 wt% Na_2_SO_4_ was carried out for 5 min, which is shorter than the coagulation time of other solvents because the hydronium ions (H3O^-^) in acidic aqueous medium can quickly diffuse into the cellulose solution and break the LiOH–urea complex shell for the regeneration of cellulose [42]. The hydrogel of regenerated cellulose was then thoroughly washed with distilled water by soaking until the pH reached the neutral region, and dried at ambient conditions for several days until constant weight was attained. RC films composed of MCC were prepared from 9 wt% of MCC–LiOH–urea aqueous mixture following the same procedure as CNF. Figure 2 represents images of RC films with a thickness of around 50 μm prepared from a 3 wt% CNF and 9 wt% MCC solution through coagulation with methanol.

### 2.3. Characterization

Chemical structures of pristine CNF, MCC, and RC films were characterized using a Fourier-transform infrared (FT-IR) spectroscopy Nicolet iS5 with iD7 ATR (Thermo Fisher Scientific K.K., Tokyo, Japan). Transmittance spectra were collected in a wavenumber range of 400–400 cm^−1^ at a resolution of 4 cm^−1^. X-ray diffraction (XRD) was performed on a diffractometer MiniFlex series (Rigaku Co., Tokyo, Japan) and operated at 40 kV and 15 mA in reflection mode with Cu Kα radiation (λ = 0.154 nm). The RC film was mounted on a sample holder and the diffraction patterns were obtained with a 2θ range from 3 to 70° at a scanning speed of 10 degrees/min along. The refinement of XRD spectra, including the breakdown between crystalline and amorphous phases, was carried out by using the MAUD Rietveld program [43]. In this study, a crystal information file (.cif) for cellulose II structure was used for performing the Rietveld method [44]. The preferential formation and orientation of crystal plane formed by intra-and inter- hydrogen bonding among regenerated cellulose crystal regions were expressed as a peak–area ratio calculated the following equation:

(1) $$\mathrm{Peak}\text{–}\mathrm{area}\mathrm{ratio}=\frac{A_{\left(110\right)}+A_{\left(020\right)}}{A_{\left(1-10\right)}}$$

where $A_{\left(110\right)}$, $A_{\left(020\right)}$, and $A_{\left(1-10\right)}$ are the peak areas of crystalline phases corresponding to (110), (020), and (1–10) planes, respectively. The tensile strength, elongation at break, and young’s modulus of RC films were evaluated by tensile testing with a tensile-compressive tester IMC-18E0 (Imoto Machinery Co., Ltd., Kyoto, Japan) at a tensile speed of 5 mm/min under room temperature. The samples were cut into the rectangular specimens with a width of 5 mm and length of 40 mm, and three specimens were tested from each film. Atomic probe microscopy (AFM) AFM5300E (Hitachi High-Tech Co., Tokyo, Japan), equipped with a silicon cantilever, was utilized to characterize the surface morphology and roughness of regenerated cellulose films. The images were recorded in dynamic force mode (DFM) under air condition. The surface morphologies of films were also observed using field emission scanning electron microscopy (FE-SEM) JSM-7800F (JEOL, Tokyo, Japan) at an accelerating voltage of 15 kV with an electron-conductive platinum layer. 

## 3. Results and Discussion

### 3.1. Chemical Structure of Regenerated Cellulose Films

**Figure 3:**
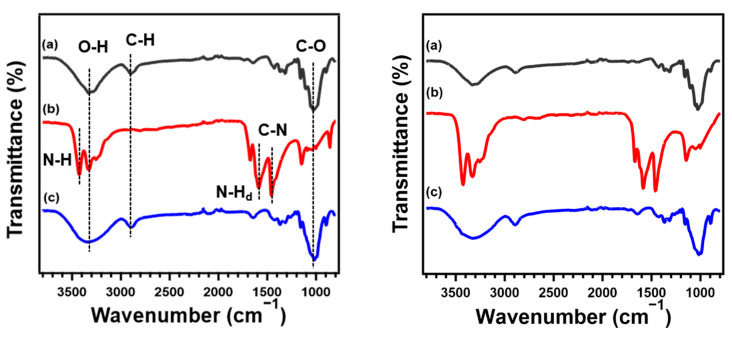
FT-IR spectra of (**a**) native cellulose, (**b**) dried mixture composed of cellulose, LiOH, and urea, and (**c**) regenerated cellulose film regenerated with methanol based on CNF (**left**) and MCC (**right**).

FT-IR/ATR spectra of RC films prepared from CNF and MCC through regeneration with methanol; a dried mixture composed of cellulose, LiOH, and urea; and the neat MCC and CNF are shown in Figure 3. The broad peak at 3000–3700 cm^−1^ is assigned to the O-H stretching vibrations arising from hydrogen bonding in molecular chains of cellulose. In addition, the spectrum of neat cellulose shows characteristic peaks at around 2900, and 1000 cm^−1^ corresponding to the stretching vibration of C-H and C-O-C, respectively. In the spectrum of the dried mixture containing cellulose, LiOH, and urea, three sharp peaks, which are attributed to N-H stretching, bending vibration of N-H, and C-N stretching of urea, are confirmed at around 3400, 1600, and 1460 cm^−1^, respectively. After regeneration and drying processes, the peaks assigned to the N-H were absent and a similar spectrum to the pristine MCC and CNF was obtained. These results provide evidence that cellulose was regenerated without derivatization after the removal of urea and LiOH through the coagulation and washing processes [45]. In a comparison of the native cellulose and RC film, the transmittance peak corresponding to the stretching vibration of O-H becomes more gradual after dissolution and coagulation. This means that the regeneration process has an effect on the intra- and inter- hydrogen bonding in cellulose. RC films coagulated with other solvents also show the same FT-IR spectra as that regenerated with methanol, implying that the functional groups of cellulose did not change depending on the species of coagulants (Figure S1). 

### 3.2. Crystal Structure of Regenerated Cellulose

**Figure 4:**
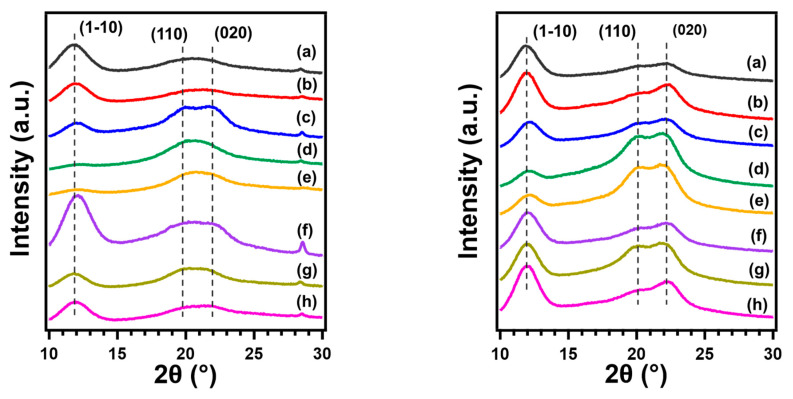
XRD spectra of regenerated cellulose films prepared from CNF (**left**) and MCC (**right**) through coagulation using (**a**) 5 wt% H_2_SO_4_ and 5 wt% Na_2_SO_4_ aqueous solution, (**b**) H_2_O, (**c**) ethylene glycol, (**d**) methanol, (**e**) ethanol, (**f**) 1-butanol, (**g**) acetone, and (**h**) *n*-hexane.

The crystal structure of RC films prepared with different types of coagulants was investigated using XRD analysis. Figure 4 depicts the XRD profiles and RC films show the structure of cellulose II which have peaks at around 2θ = 12.5°, 20°, and 22.5°, attributing to the (1–10), (110), and (020) crystal plane, respectively, while pristine cellulose is classified as cellulose I_α_ or I_β_ [46]. 

Based on the resulting XRD profiles, the relative intensities show differences for each RC film. The intensity of peak corresponding to (1–10) plane, which is formed by the arrangement of the planar glucan chains via hydrophobic interactions, became stronger than other peaks, as RC films were coagulated with H_2_O, ethylene glycol, which possesses high polarity and the low polar solvents (such as 1-butanol, acetone, and *n*-hexane). In contrast, RC films, regenerated using ethanol and methanol as coagulants, show that the intensity of the peaks corresponding to (110) and (020) planes, which are formed by inter-and intra- hydrogen bonding, are higher. These results may indicate that the preferred orientation of the crystals was changed during the process of regeneration depending on the types of coagulants. Similar behavior was observed for regenerated cellulose gels [39]. To demonstrate that preferred orientation is the cause of intensity variation, the RC film sample was cut into small particles with a mil, sieved to obtain a fine powder, and presented to the diffractometer. The XRD profiles of RC film and powdered sample are shown in Figure S2. The result shows that the diffraction pattern approaches the ideal patterns of cellulose II described in the previous work [47].

**Figure 5:**
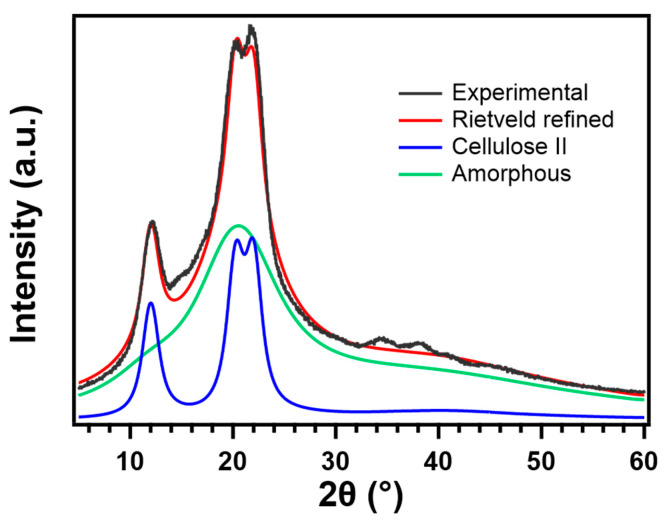
Measurement of the diffractogram performed on RC film prepared from MCC through coagulation with ethanol (black line), calculated total signal (red line), (simulated cellulose II signal (blue line), and simulated amorphous phase (green line).

For further investigation, the XRD spectra of each RC film were analyzed with the Rietveld method, and the peak areas composed of (1–10) and overlapping peaks of (110) and (020) were calculated, and then the changes in the preferred orientation of the crystal planes were evaluated as a peak–area ratio. Figure 5 depicts the result of refinement performed on the XRD pattern of RC film prepared from MCC through coagulation with ethanol as an example. The calculated peak–area ratio of the crystal plane formed intra- and inter- hydrogen bonding in RC film coagulated with high polar organic solvents, such as ethanol and methanol is higher than those coagulated with other solvents (Table 2). A possible mechanism of the structure formation of regenerated cellulose from solution has been proposed based on molecular dynamics (MD) simulations as follows [48]. First, glucopyranose rings are stacked by hydrophobic interactions to form a sheet structure. Second, these sheet structures are progressively stacked by hydrogen bonds to form thin planar crystals incorporating amorphous regions. At last, the random dispersal of these structural units in solution make contact with each other and adhere together to form three-dimensional structures. The change in crystal orientation seen in this study with the types of coagulants could be explained by the proposed mechanism and the relative diffusion rates between coagulant and LiOH–urea solution [38]. The rapid coagulation by H_2_O which possesses high polarity or acidic solution gives the cellulose chains less time to form themselves into an ordered structure that are tightly stacked by hydrogen bonding without any defects, leading to a lower peak–area ratio of (110) and (020) crystal planes. 

### 3.3. Mechanical Properties of Regenerated Celluloe Films

**Figure 6:**
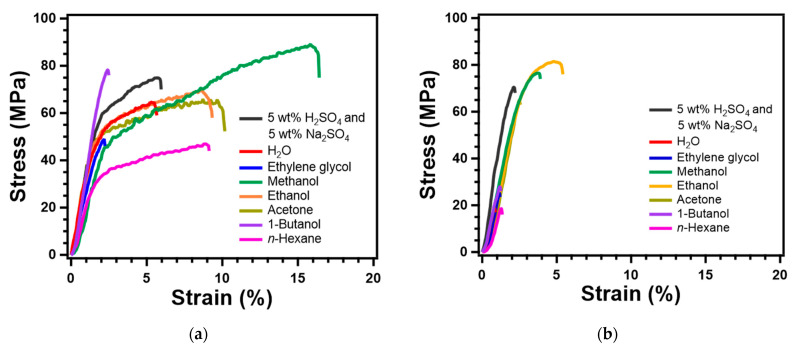
Stress-strain curves of regenerated cellulose films prepared from (**a**) CNF and (**b**) MCC through coagulation with various solvents.

Given the changes in the crystal structure of RC films depending on the species of coagulant, the obtained RC films were expected to be different in mechanical properties as well. Thus, a tensile test was conducted to evaluate the mechanical properties of RC film prepared with various coagulants. Figure 6 depicts the stress–strain curves for each RC film prepared from CNF and MCC, and the average of tensile strength, elongation at break, and young’s modulus are summarized in Table S1. The mechanical properties of RC films changed significantly depending on the solvent used as coagulants. In particular, the mechanical properties of MCC-based RC films were strongly affected by the types of coagulants. For instance, the RC film, prepared from 9 wt% MCC in LiOH–urea–H_2_O through regeneration with ethanol, shows the excellent tensile strength of 81.31 MPa and elongation at a break of 4.15%. On the other hand, MCC-based RC film coagulated with low polar solvents, such as 1-butanol and *n*-hexane, is very brittle and fragile. As compared with CNF, the elongation at break of RC films prepared from MCC resulted in lower as a whole due to the difference in aspect ratio between fibrillated cellulose and crystal. However, the tensile strength of MCC-based RC films regenerated with polar organic solvents is as high as that prepared from CNF. These results prove that MCC was packed and formed a fine structure through crystallization during dissolution and coagulation processes.

### 3.4. Correlation between Polarity of Coagulants and Properties of Regenerated Cellulose

**Figure 7:**
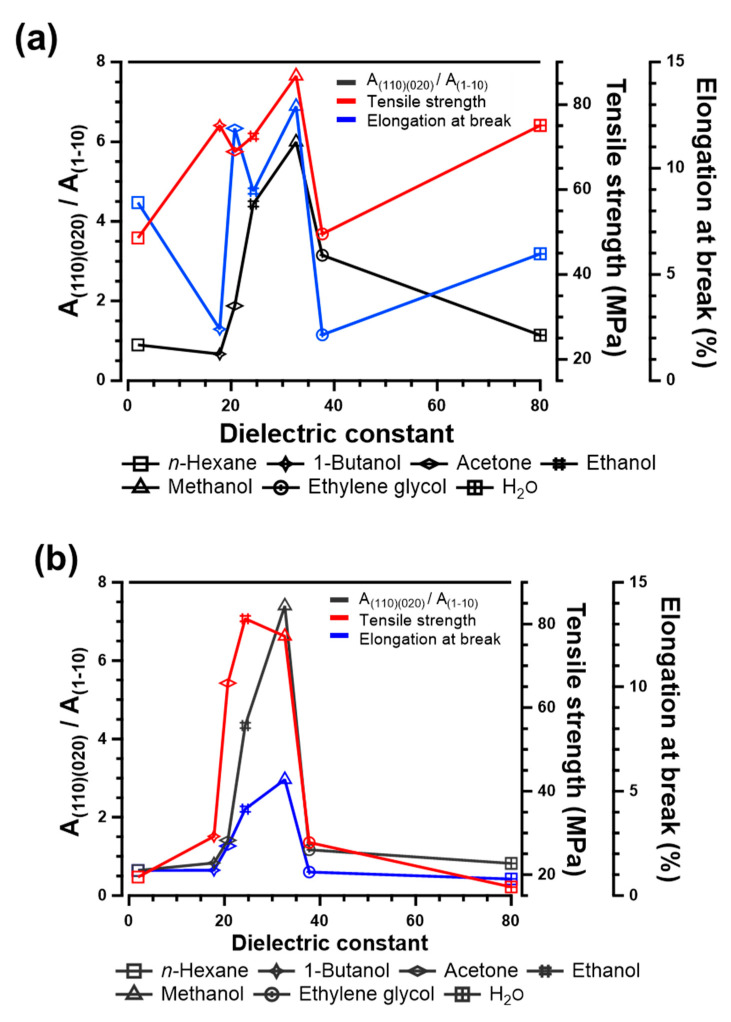
Crystal occupancy and mechanical properties of regenerated cellulose films prepared from (**a**) CNF and (**b**) MCC as a function of coagulants dielectric constant.

To control the properties of RC film by preparation condition, clarification and visualization of correlation must be crucial. In this study, the polarity of coagulants, which can be represented in dielectric constant, was adopted as a parameter to investigate the correlation between the mechanical properties and crystal structure of RC films. Figure 7 depicts the peak–area ratio of (110) and (020) lattice planes formed by intra- and inter-hydrogen bonding, to (1–10) plane, tensile strength, and elongation at break as a function of the dielectric constant of coagulants. Both RC films prepared from CNF and MCC show superior mechanical properties, as methanol which has a dielectric constant of 32.6 is used for coagulation. Moreover, the peak–area ratio of (110) and (020) crystal planes formed in RC films regenerated with methanol is much higher than RC films coagulated using other solvents. Therefore, there is an apparent correlation between the crystal and mechanical properties of RC films, and the dielectric constant of coagulants. These results revealed the correlation that the polarity of solvent has a strong effect on the interactions among the molecular chains of cellulose during the regeneration process. In addition, the correlation for RC films prepared from CNF is less apparent compared to MCC. This may be because fibrillated cellulose molecular physically intertangle during the dissolution and coagulation process, due to its high aspect ratio and molecular chain length of CNF. 

### 3.5. Surface Morphology and Roughness of Regenerated Cellulose Films

**Figure 8:**
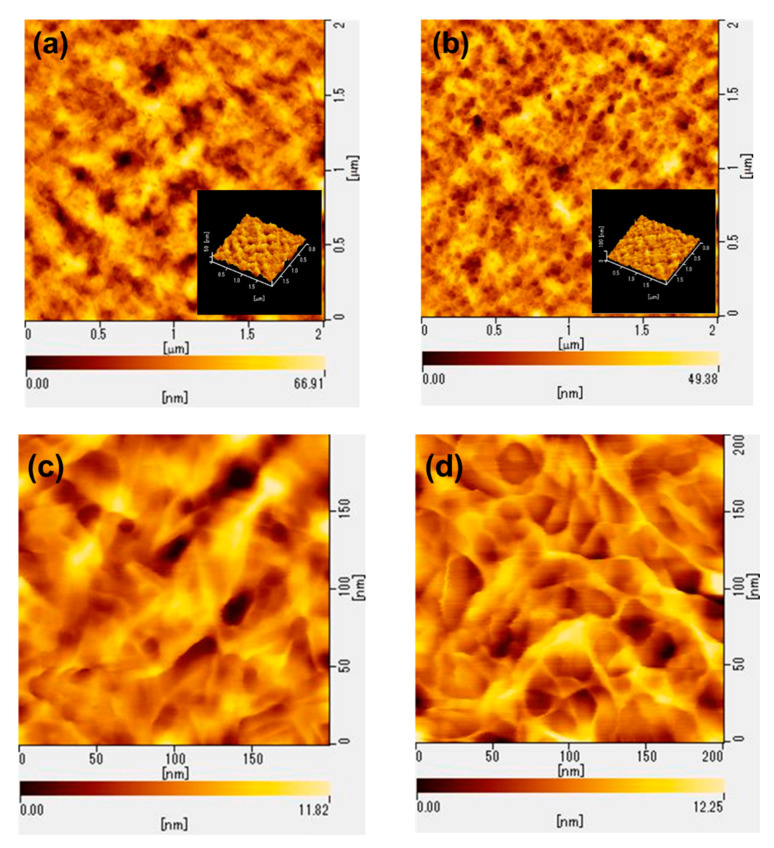
AFM images of regenerated cellulose films prepared from CNF through coagulation with (**a**,**c**) 5 wt% H_2_SO_4_ and 5 wt% Na_2_SO_4_ aqueous solution, (**b**,**d**) methanol.

It is well known that the types of coagulants not only affect mechanical properties but also the surface morphology of regenerated cellulose. In this study, AFM observation was conducted to evaluate the surface morphology and roughness of RC films prepared from 3 wt% CNF solution in LiOH–urea–H_2_O through coagulation with a mixed aqueous solution comprising 5 wt% H_2_SO_4_, 5 wt% Na_2_SO_4_, and methanol. Both RC films show nanoporous structure; however, there is an obvious difference between the RC films coagulated using the aqueous acid solution and organic solvent. The former has an agglomerated structure of cellulose fiber (Figure 8a,c), while the latter shows the fibrous structure (Figure 8b,e). The arithmetical mean height of RC film coagulated using 5 wt% H_2_SO_4_ and 5 wt% Na_2_SO_4_ aqueous solution and methanol was calculated based on AFM images and resulted in 8.05 and 5.77 nm, respectively. The SEM images also showed that the RC film prepared through coagulation with acidic aqueous solution exhibited a slightly rough surface compared to RC film regenerated with methanol (Figure S3). This indicates that polar organic solvent penetrates slowly into the cellulose solution to remove LiOH and urea, and then cellulose molecular chains self-aligned sufficiently. On the other hand, aqueous acid solution diffuses into cellulose solution rapidly and destroys the LiOH–urea complex immediately. Therefore, cellulose chains cannot form the perfect fibrillated structure and agglomerate each other. This loose structure of RC film coagulated with 5 wt% H_2_SO_4_ and 5 wt% Na_2_SO_4_ aqueous solution leads to the relatively poor mechanical performance. In contrast, the film coagulated with methanol showed the high tensile strength and elongation at break, owing to its homogeneous aggregates.

## 4. Conclusions

A series of RC films were prepared from the CNF and MCC solution through the coagulation with 5 wt% H_2_SO_4_ aqueous solution containing 5 wt% Na_2_SO_4_, distilled water, ethylene glycol, methanol, ethanol, 1-butanol, acetone, and *n*-hexane. The correlation between the polarity of solvents used for coagulation and the crystal structure as well as mechanical properties of RC films was revealed successfully. It is found that the types of coagulants affect the orientation of the crystal plane formed by intra- and inter- hydrogen bonding, which is described as (110) and (020) lattice planes. The experimental results demonstrated that RC films prepared from 3 wt% CNF and 9 wt% MCC solution through regeneration using high-polarity organic solvents, such as methanol and ethanol showed a preferential orientation to the (110) and (020) planes, and higher tensile strength and elongation at break compared to the aqueous solution and low polar solvents. The change in the mechanical performance and structure of the RC films caused by the polarity of the coagulant can be explained as follows. When the cellulose solution was coagulated with acidic aqueous solution or water, the coagulant rapidly penetrates and neutralizes the cellulose solution, leading to the enforced agglomeration of cellulose chains and loose structure with low crystal orientation and mechanical properties. On the other hand, the coagulation with high-polarity organic solvents enables cellulose chains to form the self-aligned structure through hydrogen bonding, owing to the slower exchange between cellulose solution and coagulant. Thus, RC films prepared with methanol and ethanol as coagulant showed higher crystal orientation and mechanical performance. In addition to the coagulation conditions, it was also found that the mechanical properties of the RC films can be altered by the morphology of cellulose. RC films prepared from CNF showed higher elongation, owing to its high aspect ratio compared to crystalline cellulose. The findings in this study will be applicable as a parameter to control the properties of regenerated cellulose film depending on its application.

## Figures and Tables

**Table 1:** Preparation conditions of regenerated cellulose film and polarity of coagulants.

Coagulant	Coagulation Time (min)	Dielectric Constant
5 wt% H_2_SO_4_ and 5 wt% Na_2_SO_4_	5	-
H_2_O	30	80.0
Ethylene glycol	30	37.7
Methanol	30	32.6
Ethanol	30	24.3
1-Butanol	30	17.8
Acetone	30	20.7
*n*-Hexane	30	1.9

**Table 2:** Crystal properties of regenerated cellulose film.

Types of Cellulose	Coagulant	Peak Area		Peak–Area Ratio (*A*_(110)(020)/(1–10)_)
		*A*_(1–10)_	*A*_(110)(020)_	
CNF	5 wt% H_2_SO_4_ and 5 wt% Na_2_SO_4_	10,706.40	3887.85	0.36
	H_2_O	6030.11	6900.22	1.14
	Ethylene glycol	3049.70	9608.20	3.15
	Methanol	2161.39	12,965.80	5.99
	Ethanol	1825.36	8105.36	4.44
	1-Butanol	13,135.90	8769.13	0.67
	Acetone	3796.31	7145.69	1.88
	*n*-Hexane	6849.58	6167.59	0.90
MCC	5 wt% H_2_SO_4_ and 5 wt% Na_2_SO_4_	10,066.60	5441.96	0.54
	H_2_O	13,018.40	10,730.86	0.82
	Ethylene glycol	7022.86	8236.69	1.17
	Methanol	3180.77	23,543.59	7.40
	Ethanol	5064.45	22,050.30	4.35
	1-Butanol	12,792.60	10,635.05	0.83
	Acetone	12,773.30	18,001.92	1.41
	*n*-Hexane	14,839.20	9526.60	0.64

## Data Availability

Data is contained within the article and Supplementary Materials.

## Supplementary Materials

The following are available online at https://www.mdpi.com/article/10.3390/polym13244450/s1, Figure S1: FT-IR spectra of regenerated cellulose film prepared from (a) CNF and (b) MCC using various solvents as a coagulant, Figure S2: XRD spectra of the pristine and powdered regenerated cellulose film prepared from MCC through coagulation with 5 wt% H_2_SO_4_ aqueous solution containing 5 wt% Na_2_SO_4_, Figure S3: SEM images of RC films prepared from CNF through coagulation with (a) 5 wt% H_2_SO_4_ aqueous solution containing 5 wt% Na_2_SO_4_ and (b) methanol, Table S1: Mechanical properties of regenerated cellulose film.

## References

[1] Schnurr R.E.J., Alboiu V., Chaudhary M., Corbett R.A., Quanz M.E., Sankar K., Srain H.S., Thavarajah V., Xanthos D., Walker T.R. (2018). Reducing marine pollution from single-use plastics (SUPs): A review. Mar. Pollut. Bull..

[2] Farah S., Anderson D.G., Langer R. (2016). Physical and mechanical properties of PLA, and their functions in widespread applications—A comprehensive review. Adv. Drug Deliv. Rev..

[3] Watanabe K., Ando Y., Shirai Y., Nishida H. (2013). A cross-linked polystyrene supported hindered lithium amide as a deprotonation reagent for α-methylation of lactic acid. Tetrahedron Lett..

[4] Eksiler K., Andou Y., Shirai Y. (2017). Green polymer blends compatibilized with biomass derived-agents. Acad. J. Environ. Sci..

[5] Niu X., Liu Y., Song Y., Han J., Pan H. (2018). Rosin modified cellulose nanofiber as a reinforcing and co-antimicrobial agents in polylactic acid /chitosan composite film for food packaging. Carbohydr. Polym..

[6] Moustafa H., El Kissi N., Abou-Kandil A.I., Abdel-Aziz M.S., Dufresne A. (2017). PLA/PBAT bionanocomposites with antimicrobial natural rosin for green packaging. ACS Appl. Mater. Interfaces.

[7] Muneer F., Rasul I., Azeem F., Siddique M.H., Zubair M., Nadeem H. (2020). Microbial Polyhydroxyalkanoates (PHAs): Efficient Replacement of Synthetic Polymers. J. Polym. Environ..

[8] Yee L.N., Mumtaz T., Mohammadi M., Phang L.Y., Ando Y., Raha A.R., Sudesh K., Ariffin H., Hassan M.A., Zakaria M.R. (2012). Polyhydroxyalkanoate Synthesis by Recombinant Escherichia coli JM109 Expressing PHA Biosynthesis Genes from Comamonas sp. EB172. J. Microb. Biochem. Technol..

[9] Mohammadi M., Hassan M.A., Phang L.Y., Ariffin H., Shirai Y., Ando Y. (2012). Recovery and purification of intracellular polyhydroxyalkanoates from recombinant Cupriavidus necator using water and ethanol. Biotechnol. Lett..

[10] Sun J., Shen J., Chen S., Cooper M.A., Fu H., Wu D., Yang Z. (2018). Nanofiller reinforced biodegradable PLA/PHA composites: Current status and future trends. Polymers.

[11] Lee J.S., Hwang G.H., Kwon Y.S., Jeong Y.G. (2020). Impacts of cellulose nanofibril and physical aging on the enthalpy relaxation behavior and dynamic mechanical thermal properties of Poly(lactic acid) composite films. Polymers.

[12] Hassanajili S., Karami-Pour A., Oryan A., Talaei-Khozani T. (2019). Preparation and characterization of PLA/PCL/HA composite scaffolds using indirect 3D printing for bone tissue engineering. Mater. Sci. Eng. C.

[13] Kim D., Andou Y., Shirai Y., Nishida H. (2011). Biomass-based composites from poly(lactic acid) and wood flour by vapor-phase assisted surface polymerization. ACS Appl. Mater. Interfaces.

[14] Lee H.S., Wakisaka M., Nagasawa N., Nishida H., Andou Y. (2014). Development of biocomposites containing erianthus arudinaceus as cellulose resource crops. Kobunshi Ronbunshu.

[15] Eksiler K., Andou Y., Ariffin H., Shirai Y. (2019). Surface modification for nano-lignocellulose fiber through vapor-phase-assisted surface polymerization. J. Polym. Sci. Part A Polym. Chem..

[16] Yasim-Anuar T.A.T., Ariffin H., Norrrahim M.N.F., Hassan M.A., Andou Y., Tsukegi T., Nishida H. (2020). Well-dispersed cellulose nanofiber in low density polyethylene nanocomposite by liquid-Assisted extrusion. Polymers.

[17] Eksiler K., Andou Y., Yilmaz F., Shirai Y., Ariffin H., Hassan M.A. (2017). Dynamically controlled fibrillation under combination of ionic liquid with mechanical grinding. J. Appl. Polym. Sci..

[18] Ye D., Lei X., Cheng Q., Chang C., Hu L., Zhang L. (2019). Ultrahigh Tough, Super Clear, and Highly Anisotropic Nanofiber-Structured Regenerated Cellulose Films. ACS Nano.

[19] Hyden W.L. (1929). Manufacture and Properties of Regenerated Cellulose Films. Ind. Eng. Chem..

[20] Klemm D., Heublein B., Fink H.P., Bohn A. (2005). Cellulose: Fascinating biopolymer and sustainable raw material. Angew. Chem. Int. Ed..

[21] Medronho B., Lindman B. (2015). Brief overview on cellulose dissolution/regeneration interactions and mechanisms. Adv. Colloid Interface Sci..

[22] Rosenau T., Potthast A., Adorjan I., Hofinger A., Sixta H., Firgo H., Kosma P. (2002). Cellulose solutions in N-methylmorpholine-N-oxide (NMMO)—Degradation processes and stabilizers. Cellulose.

[23] Zhao H., Kwak J.H., Wang Y., Franz J.A., White J.M., Holladay J.E. (2007). Interactions between cellulose and N-methylmorpholine-N-oxide. Carbohydr. Polym..

[24] Rosenau T., Potthast A., Sixta H., Kosma P. (2001). The chemistry of side reactions and byproduct formation in the system NMMO/cellulose (Lyocell process). Prog. Polym. Sci..

[25] Swatloski R.P., Spear S.K., Holbrey J.D., Rogers R.D. (2002). Dissolution of cellose with ionic liquids. J. Am. Chem. Soc..

[26] Xu A., Wang J., Wang H. (2010). Effects of anionic structure and lithium salts addition on the dissolution of cellulose in 1-butyl-3-methylimidazolium-based ionic liquid solvent systems. Green Chem..

[27] Vitz J., Erdmenger T., Haensch C., Schubert U.S. (2009). Extended dissolution studies of cellulose in imidazolium based ionic liquids. Green Chem..

[28] Zavrel M., Bross D., Funke M., Büchs J., Spiess A.C. (2009). High-throughput screening for ionic liquids dissolving (ligno-)cellulose. Bioresour. Technol..

[29] Raut D.G., Sundman O., Su W., Virtanen P., Sugano Y., Kordas K., Mikkola J.P. (2015). A morpholinium ionic liquid for cellulose dissolution. Carbohydr. Polym..

[30] Pang J.-H., Liu X., Wu M., Wu Y.-Y., Zhang X.-M., Sun R.-C. (2014). Fabrication and characterization of regenerated cellulose films using different ionic liquids. J. Spectrosc..

[31] Zhou J., Zhang L. (2000). Solubility of cellulose in NaOH/Urea Aqueous Solution. Polym. J..

[32] Cai J., Zhang L. (2005). Rapid dissolution of cellulose in LiOH/urea and NaOH/urea aqueous solutions. Macromol. Biosci..

[33] Jiang Z., Fang Y., Xiang J., Ma Y., Lu A., Kang H., Huang Y., Guo H., Liu R., Zhang L. (2014). Intermolecular interactions and 3D structure in cellulose-NaOH-urea aqueous system. J. Phys. Chem. B.

[34] Bingbing W., Bing L., Jie X., Li C.Y. (2008). Hierarchically ordered polymer nanofibers via electrospinning and controlled polymer crystallization. Macromolecules.

[35] Yang G., Miyamoto H., Yamane C., Okajima K. (2007). Structure of regenerated cellulose films from cellulose/aqueous NaOH solution as a function of coagulation conditions. Polym. J..

[36] Yamane C., Mori M., Saito M., Okajima K. (1996). Structures and mechanical properties of cellulose filament spun from cellulose/aqueous NaOH solution system. Polym. J..

[37] Mazlan N.S.N., Zakaria S., Gan S., Hua C.C., Baharin K.W. (2019). Comparison of regenerated cellulose membrane coagulated in sulphate based coagulant. Cerne.

[38] Zhang L., Mao Y., Zhou J., Cai J. (2005). Effects of coagulation conditions on the properties of regenerated cellulose films prepared in NaOH/Urea aqueous solution. Ind. Eng. Chem. Res..

[39] Isobe N., Kim U.J., Kimura S., Wada M., Kuga S. (2011). Internal surface polarity of regenerated cellulose gel depends on the species used as coagulant. J. Colloid Interface Sci..

[40] Yamane C., Aoyagi T., Ago M., Sato K., Okajima K., Takahashi T. (2006). Two different surface properties of regenerated cellulose due to structural anisotropy. Polym. J..

[41] Yang Q., Fukuzumi H., Saito T., Isogai A., Zhang L. (2011). Transparent cellulose films with high gas barrier properties fabricated from aqueous alkali/urea solutions. Biomacromolecules.

[42] Zhu K., Qiu C., Lu A., Luo L., Guo J., Cong H., Chen F., Liu X., Zhang X., Wang H. (2018). Mechanically strong multifilament fibers spun from cellulose solution via inducing formation of nanofibers. ACS Sustain. Chem. Eng..

[43] Lutterotti L. (2010). Total pattern fitting for the combined size-strain-stress-texture detrmination in thin film diffraction. Nucl. Instrum. Methods B.

[44] Langan P., Nishiyama Y., Chanzy H. (2001). X-ray structure of mercerized cellulose Ⅱ at 1 Å resolution. Biomacromolecules.

[45] Wei Q.-Y., Lin H., Yang B., Li L., Zhang L.-Q., Huang H.-D., Zhong G.-J., Xu L., Li Z.-M. (2020). Structure and properties of all-cellulose composites prepared by controlling the dissolution temperature of a NaOH/Urea solvent. Ind. Eng. Chem..

[46] Yamane C. (2015). Structure formation of regenerated cellulose from its solution and resultant features of high wettability: A review. Nord. Pulp Pap. Res. J..

[47] French A.D. (2014). Idealized powder diffraction patterns for cellulose polymorphs. Cellulose.

[48] Miyamoto H., Umemura M., Aoyagi T., Yamane C., Ueda K., Takahashi K. (2009). Structural reorganization of moleular sheets derived from cellulose Ⅱ by molecular dynamics simulations. Carbohydr. Res..

