# Supplementary material for: The Relationship between Crystal Structure and Mechanical Performance for Fabrication of Regenerated Cellulose Film through Coagulation Conditions
Source: Polymers (Basel). 2021 Dec 18;13(24):4450. doi: 10.3390/polym13244450 (PMC8708383; doi:10.3390/polym13244450)
Supplement: Supplementary file 1 [file polymers-13-04450-s001.zip › polymers-1485750-supplementary.pdf]

polymers

MDPI

Supplementary Materials

The Relationship between Crystal Structure and Mechanical Performance for Fabrication of Regenerated Cellulose Film through Coagulation Conditions

Tessei Kawano [1], Satoshi Iikubo [1] and Yoshito Andou [1,2,*]

[1] Department of Life Science and Systems Engineering, Graduate School of Life Science and Systems Engineering, Kyushu Institute of Technology, 2-4 Hibikino, Wakamatsu, Kitakyushu 808-0196, Fukuoka, Japan; kawano.tessei758@mail.kyutech.jp (T.K.); iikubo@life.kyutech.ac.jp (S.I.)

[2] Department of Advanced Materials Science and Engineering, Faculty of Engineering Sciences, Kyushu University, Kasuga 816-8580, Fukuoka, Japan

[3] Collaborative Research Centre for Green Materials on Environmental Technology, Kyushu Institute of Technology, 2-4 Hibikino, Wakamatsu, Kitakyushu 808-0196, Fukuoka, Japan

* Correspondence: yando@life.kyutech.ac.jp

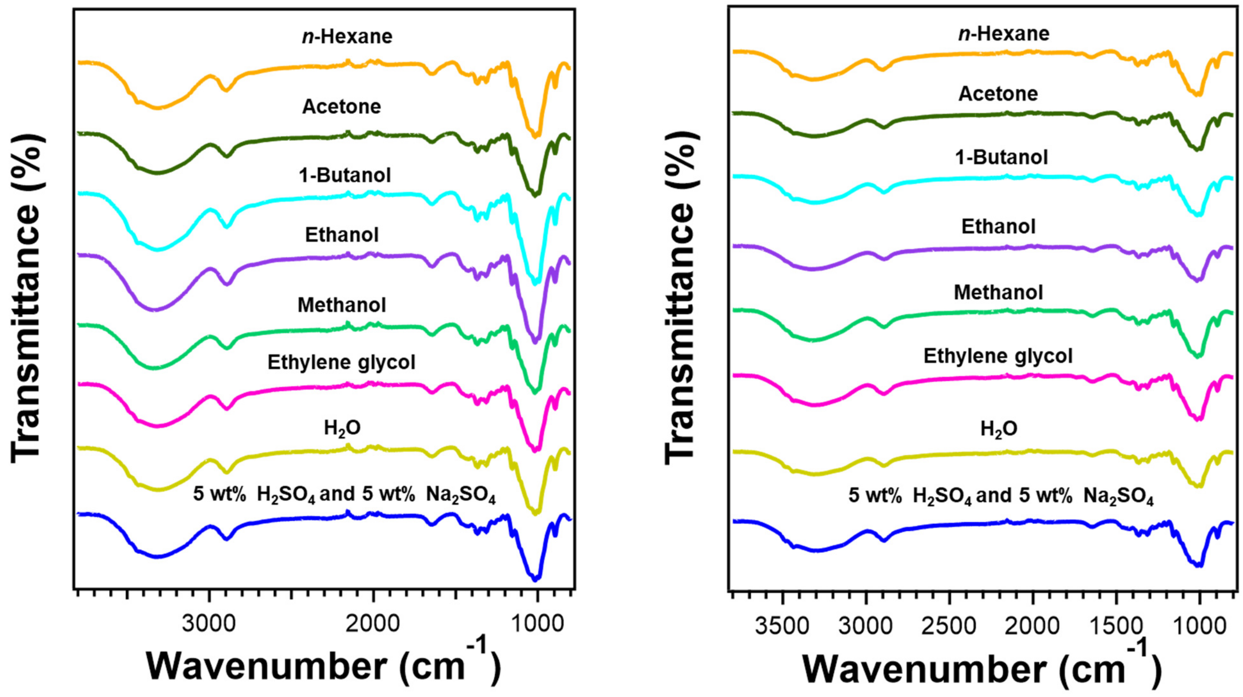

(**a**) (**b**)

Figure S1. FT-IR spectra of regenerated cellulose film prepared from (a) CNF and (b) MCC using various solvents as a coagulant.

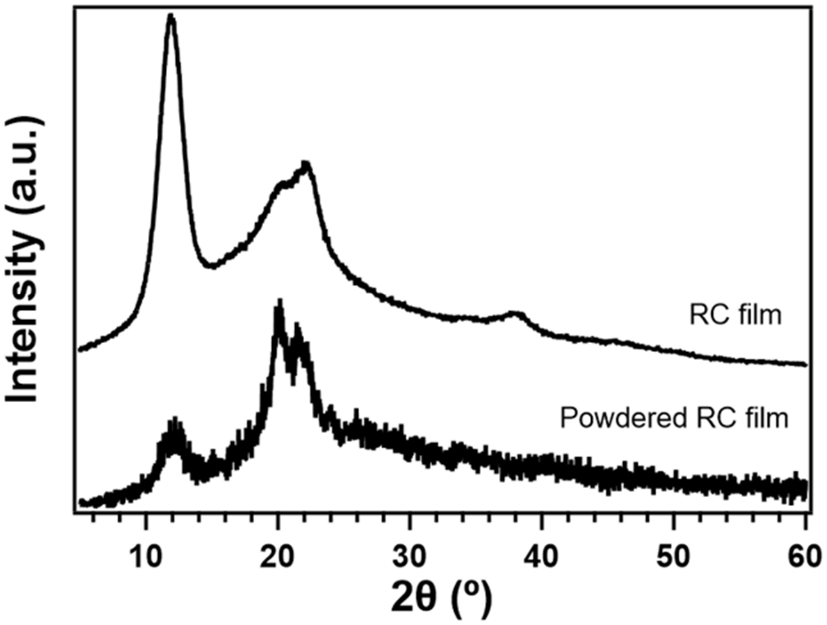

Figure S2. XRD spectra of the pristine and powdered regenerated cellulose film prepared from MCC through coagulation with 5 wt% H_2SO_4 aqueous solution containing 5 wt% Na_2SO_4.

Table S1. Mechanical properties of regenerated cellulose film.

Cellulose	Coagulant	Tensile strength (MPa)	Elongation at break (%)	Young's modulus (GPa)
CNF	5 wt% H_2SO_4 and 5 wt% Na_2SO_4	75.95 ± 1.38	4.69 ± 0.93	3.96 ± 0.35
	H_2O	75.09 ± 7.55	5.97 ± 1.84	3.91 ± 0.25
	Ethylene glycol	49.54 ± 4.72	2.16 ± 0.12	2.66 ± 0.51
	Methanol	86.75 ± 6.69	12.91 ± 2.76	2.94 ± 0.45
	Ethanol	72.57 ± 9.95	8.92 ± 0.84	3.39 ± 0.10
	1-Butanol	75.09 ± 9.09	2.43 ± 0.45	3.58 ± 0.13
	Acetone	68.89 ± 2.10	11.88 ± 1.24	3.53 ± 0.19
	n-Hexane	48.59 ± 4.37	8.39 ± 2.03	2.37 ± 0.12
MCC	5 wt% H_2SO_4 and 5 wt% Na_2SO_4	75.85 ± 12.14	2.30 ± 0.36	4.28 ± 0.79
	H_2O	17.04 ± 4.25	0.79 ± 0.10	1.89 ± 0.49
	Ethylene glycol	27.70 ± 9.85	1.13 ± 0.11	2.20 ± 1.15
	Methanol	77.08 ± 8.38	5.56 ± 2.04	2.65 ± 0.22
	Ethanol	81.31 ± 6.32	4.15 ± 0.95	3.21 ± 0.27
	1-Butanol	29.21 ± 4.78	1.22 ± 0.15	2.44 ± 0.23
	Acetone	65.83 ± 5.98	2.38 ± 0.25	3.25 ± 0.88
	n-Hexane	19.42 ± 2.24	1.20 ± 0.11	1.04 ± 0.17

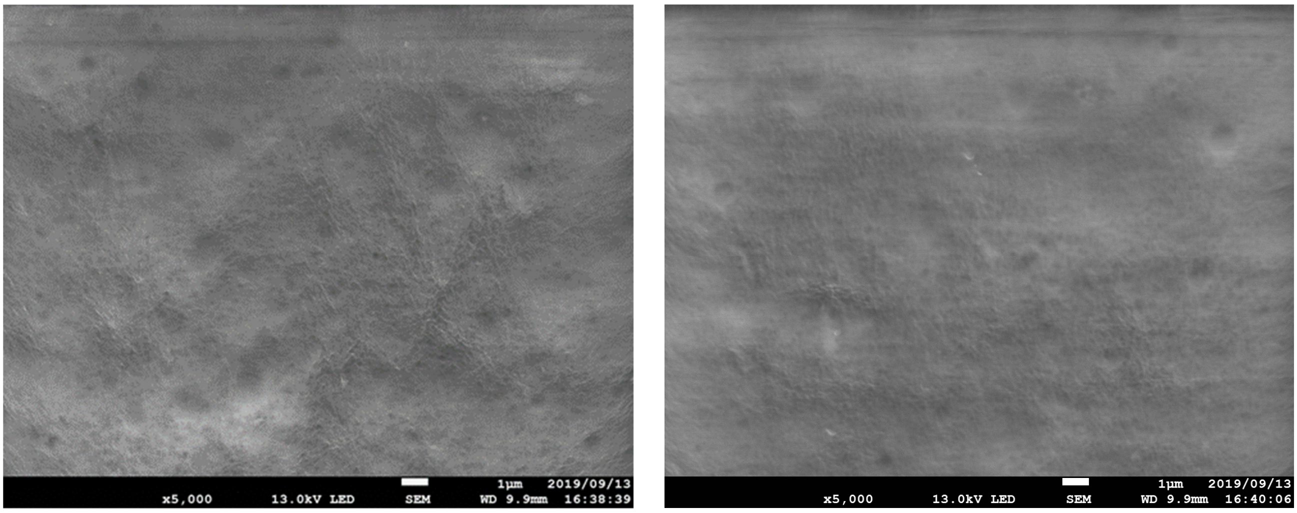

(**a**) (**b**)

Figure S3. SEM images of RC films prepared from CNF through coagulation with (a) 5 wt% H2SO4 aqueous solution containing 5 wt% Na2SO4 and (b) methanol.
